# Differences in the perception of stigma in schizophrenia between men and women: a brief qualitative approach

**DOI:** 10.1192/j.eurpsy.2024.1679

**Published:** 2024-08-27

**Authors:** P. Andres-Olivera, B. Arribas-Simon, E. D. Alvarez, B. Bote, C. Martin-Gomez, C. Payo, C. Munaiz, R. Brito, M. Ligero-Argudo

**Affiliations:** ^1^Psychiatric Service, University of Salamanca Health Complex; ^2^Psychiatric Unit. School of Medicine, University of Salamanca; ^3^PRINT, Biomedical Research Institute (IBSAL), Salamanca; ^4^Psychiatric Service, University of Valladolid Healthcare Complex, Valladolid, Spain

## Abstract

**Introduction:**

Men and women with psychosis have different courses and presentations of symptoms. Men with psychosis have an earlier onset of illness, more negative symptoms, and worse premorbid functioning. Women, on the other hand, have better social functioning and less substance abuse. Despite these evident differences, there are few studies that delve into these distinctions, especially from a subjective perspective.

**Objectives:**

The aim of this study is to understand the differences in the perception of psychosis between men and women.

**Methods:**

Five women and five men diagnosed with schizophrenia participated in the study. They were matched so that the age difference between them was no more than 5 years, with ages ranging from 40 to 56 years. Participants had not experienced acute decompensation of their underlying illness and had not required admission to an Acute Care Unit in the 6 months prior to inclusion in the study. Data collection was conducted through the Spanish translation of the Indiana Psychiatric Illness Interview, consisting of five parts: a narrative about their life, a narrative about the illness, questions related to how the illness has changed their life and what has not changed, the overall influence of the illness on their life, and lastly, expectations for the future.

**Results:**

Men expressed more concerns about work (4 men versus 2 women), while women expressed more concerns about not having become mothers (3 out of 5 women, compared to one man). All participants shared experiences of isolation in intimate relationships, including romantic relationships. Regarding stigma, three women believed that people treated them like children and dismissed their opinions. However, two of them viewed this behavior from their loved ones positively. Two women discussed the impact that psychosis and medications had on their bodies and how others had reacted to these changes

**Conclusions:**

The concerns and stigma associated with mental illness differ between genders. These differences should be taken into account when developing specific biopsychosocial treatment plans.

**Disclosure of Interest:**

None Declared

